# A New Twist: The Combination of Sulbactam/Avibactam Enhances Sulbactam Activity against Carbapenem-Resistant *Acinetobacter baumannii* (CRAB) Isolates

**DOI:** 10.3390/antibiotics10050577

**Published:** 2021-05-13

**Authors:** Fernando Pasteran, Jose Cedano, Michelle Baez, Ezequiel Albornoz, Melina Rapoport, Jose Osteria, Sabrina Montaña, Casin Le, Grace Ra, Robert A. Bonomo, Marcelo E. Tolmasky, Mark Adams, Alejandra Corso, Maria Soledad Ramirez

**Affiliations:** 1 National/Regional Reference Laboratory for Antimicrobial Resistance (NRL), Servicio Antimicrobianos, Instituto Nacional de Enfermedades Infecciosas, ANLIS “Dr. Carlos G. Malbrán”, C1282AFF Buenos Aires, Argentina or fpasteran@anlis.gob.ar (F.P.); Ealbornoz@anlis.gob.ar (E.A.); Rapoport@anlis.gob.ar (M.R.); Acorso@anlis.gob.ar (A.C.); 2 Center for Applied Biotechnology Studies, Department of Biological Science, College of Natural Sciences and Mathematics, California State University Fullerton, Fullerton, CA 92831-3599, USA; Jcedano@csu.fullerton.edu (J.C.); michelleb777@csu.fullerton.edu (M.B.); josteria@csu.fullerton.edu (J.O.); casle@Fullerton.edu (C.L.); gracera@csu.fullerton.edu (G.R.); mtolmasky@fullerton.edu (M.E.T.); 3 Laboratorio de Bacteriología Clínica, Departamento de Bioquímica Clínica, Hospital de Clínicas José de San Martín, Facultad de Farmacia y Bioquímica, C1120 Buenos Aires, Argentina; sabri.mon@hotmail.com; 4 Research Service and GRECC, Louis Stokes Cleveland Department of Veterans Affairs Medical Center, Cleveland, OH 44106, USA; Robert.Bonomo@va.gov; 5 Departments of Medicine, Pharmacology, Molecular Biology and Microbiology, Biochemistry, Proteomics and Bioinformatics, Case Western Reserve University School of Medicine, Cleveland, OH 44106, USA; 6 CWRU-Cleveland VAMC Center for Antimicrobial Resistance and Epidemiology (Case VA CARES), Cleveland, OH 44106, USA; 7 The Jackson Laboratory, Farmington, CT 06032, USA; madams@jcvi.org

**Keywords:** *Acinetobacter*, carbapenem-resistance, sulbactam, avibactam, relebactam

## Abstract

An increasing number of untreatable infections are recorded every year. Many studies have focused their efforts on developing new β-lactamase inhibitors to treat multi-drug resistant (MDR) isolates. In the present study, sulbactam/avibactam and sulbactam/relebactam combination were tested against 187 multi-drug resistant (MDR) *Acinetobacter* clinical isolates; both sulbactam/avibactam and sulbactam/relebactam restored sulbactam activity. A decrease ≥2 dilutions in sulbactam MICs was observed in 89% of the isolates when tested in combination with avibactam. Sulbactam/relebactam was able to restore sulbactam susceptibility in 40% of the isolates. In addition, the susceptibility testing using twenty-three *A. baumannii* AB5075 knockout strains revealed potential sulbactam and/or sulbactam/avibactam target genes. We observed that diazabicyclooctanes (DBOs) β-lactamase inhibitors combined with sulbactam restore sulbactam susceptibility against carbapenem-resistant *Acinetobacter* clinical isolates. However, relebactam was not as effective as avibactam when combined with sulbactam. Exploring novel combinations may offer new options to treat *Acinetobacter* spp. infections, especially for widespread oxacillinases and metallo-β-lactamases (MBLs) producers.

## 1. Introduction

Infections caused by antibiotic-resistant bacteria are increasing in frequency, resulting in significant patient morbidity and mortality [1,2]. *Acinetobacter baumannii* is a nosocomial pathogen frequently resistant to multiple drugs. *A. baumannii* causes pneumonia, bacteremia, and wound infections with associated high mortality rates. The most problematic strains are those resistant to carbapenems (carbapenem-resistant *A. baumannii,* CRAB). In most cases, resistance is caused by either chromosomal or plasmid-mediated class D oxacillinases (*bla*_OXA_) [3,4,5,6,7,8,9], but recently, *bla*_NDM-1_ has also been increasingly observed [8,10,11,12,13,14,15]. The advent of CRAB left few active antimicrobials, and unfortunately, these “last resort” antibiotics (polymyxins, tigecycline, minocycline, and amikacin) are limited in efficacy and toxic [16]. Interestingly, sulbactam, a β-lactamase inhibitor of Ambler class A enzymes, exhibits an inherent antibacterial activity against some bacterial species, including *Neisseria gonorrhoeae, Bacteroides fragilis,* and *Acinetobacter* spp. [17]. Although ampicillin-sulbactam effectively treated pneumonia, bacteremia, and other nosocomial infections caused by *A. baumannii*, increasing resistance to this combination is becoming ever more common [18,19,20].

Many recent studies focused on the development of new β-lactamase inhibitors to treat multi-drug resistant (MDR) infections [21,22,23,24,25,26,27,28]. This research resulted in the design of important types of β-lactamase inhibitors that include diazabicyclooctanes (DBOs) and boronate-based compounds [25,29]. A promising DBO inhibitor is avibactam, a class A and C β-lactamase inhibitor with some activity against class D enzymes (OXA-48-like) [25,27,30,31]. While this inhibitor showed promising activity in combination with ceftazidime to treat infections caused by pathogens with extreme resistance, it failed against *Acinetobacter* [28]. In addition, a new FDA-approved DBO, relebactam, was effective in combination with imipenem against KPC carbapenemases, AmpCs (class C), and/or extended-spectrum β-lactamases (ESBLs) produced by *Enterobacterales* [24,26,30].

On the other hand, sulbactam combined with avibactam exhibited a synergistic activity against class D carbapenemase-producing *A. baumannii* isolates [31]. Previous studies evaluated the activity of sulbactam combined with other antibiotics [32,33,34,35]. However, the performance of the combination of sulbactam with avibactam or relebactam against carbapenem-resistant *Acinetobacter* is unknown. By interacting with multiple penicillin binding proteins and β-lactamases, we hypothesized that the combination of sulbactam and avibactam or relebactam further sensitizes *A. baumannii* and reduces MICs.

## 2. Results

**Figure 1:**
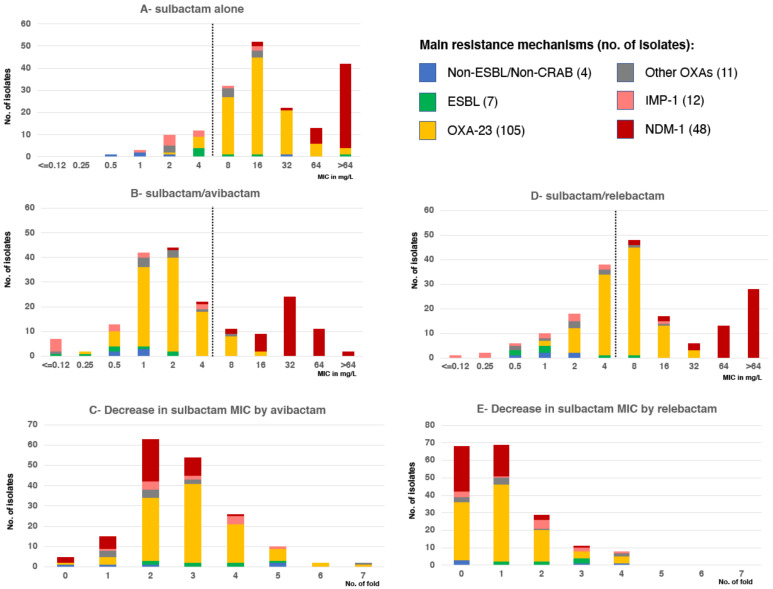
MIC frequency distribution of sulbactam/avibactam, sulbactam/relebactam and sulbactam alone according to the main resistance mechanism. Number of isolates with the indicated sulbactam (**A**) sulbactam/avibactam (**B**) or sulbactam/relebactam (**D**) MIC value, according to the resistant mechanisms. No. isolates with the indicated concentration decrease in sulbactam MIC by addition of avibactam (**C**) or relebactam (**E**) according to the resistant mechanisms. The dotted line indicates the breakpoint value that defines susceptible/resistant (**A**,**B**,**D**) according to definitions.

Of the 187 selected *Acinetobacter* strains, including non-ESBL/non-CRAB (n = 4), ESBL (n = 7 as follows, 5 PER-2, 1 VEB-1, 1 CTX-M-15), OXA-23 (n = 105), OXA-58 (n = 8), OXA-143 (n = 2), IS*Aba1-bla*_OXA-66_ (n = 1), IMP-1 (n = 12) and NDM-1 (n = 48) producers, 26/187 (14%) were inhibited by ≤4 mg/L of sulbactam alone. Only 10/116 (9%) of OXA producers and none of the NDM producers were susceptible to sulbactam (MIC ≤ 4 mg/L) (Figure 1A). Narrow-spectrum β-lactamases such as TEM-1, a common contributor to sulbactam resistance in *Acinetobacter* (19), were detected only in 9 isolates; 8 isolates also produced OXA-23 and one OXA-58. Co-production of ESBL among CRAB, another attributable cause of sulbactam resistance, was observed in 17 isolates: PER-7 and PER-2 ESBL were detected among 14 NDM and 3 OXA-58 producers, respectively. The MICs of sulbactam showed a bi-modal distribution with a first mode of 16 mg/L, corresponding to OXA producers, and a second mode presumable above the highest evaluated value (>64 mg/L) due to NDM producers (Figure 1A).

### 2.1. Sulbactam/Avibactam Susceptibility Testing of Acinetobacter spp. Clinical Strains

We observed that 129/187 (69%) *Acinetobacter* spp. isolates were inhibited by 4 mg/L of sulbactam when combined with 4 mg/L avibactam (Figure 1B). Remarkably, 105/116 (91%) of OXA producers showed MIC equal to or less than 4 mg/L for the sulbactam/avibactam combination. NDM producers showed a reduction in the MICs of sulbactam due to the addition of avibactam, although MIC values were above 4 mg/L for all but one isolate (Figure 1B). The MICs of sulbactam/avibactam also showed a bi-modal distribution but with its modes displaced toward lower MICs. The first mode of 2 mg/L (3-fold lower when compared to sulbactam alone) corresponded to OXA producers, and the second mode of 32 mg/L to NDM producers. The MIC_50_ and MIC_90_ of sulbactam/avibactam were 2 and 4 mg/L for OXA producers compared to 16 and 64 mg/L for sulbactam alone, respectively. Among NDM producers, the MIC_50_ and MIC_90_ values of >64 mg/L for sulbactam alone decreased to 32 and 64 mg/L for sulbactam/avibactam, respectively. An experiment using an expanded range of concentrations using as highest value 1024 mg/L on a subset of NDM producers (n = 40/48) showed an MIC_50_ of 256 mg/L for sulbactam alone and 32 mg/L for sulbactam/avibactam. This result indicated that there is an enhancement of sulbactam activity by avibactam in this group of strains.

The average sulbactam MIC decrease (range) by avibactam was: 3-fold decrease (0–5) for non-ESBL/non-CRE, 4-fold decrease (2–9) for ESBL, 3-fold decrease (0–7) for OXA-23, 2.4-fold decrease (2–7) for other OXAs, 2.4-fold decrease (1–5) for IMP-1 and 2-fold decrease (0–4) for NDM producers (Figure 1C).

**Figure 2:**
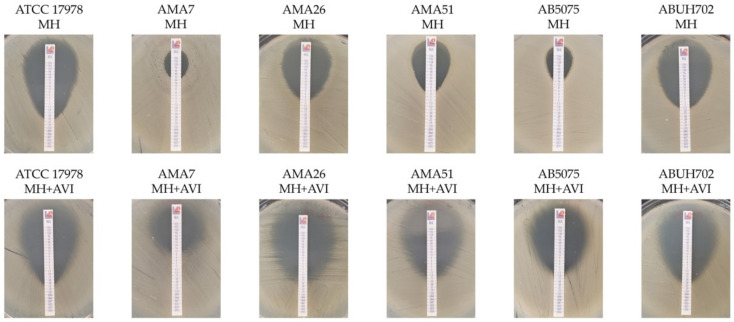
Gradient diffusion (E-test) of sulbactam/avibactam (MH + AVI) and sulbactam (MH) in selected representative strains.

A total of 159 (89%) of the strains showed an equal or greater than twofold decrease in the sulbactam MIC by avibactam: 32/40 (80%) NDMs, 11/12 (92%) IMP-1, 106/116 (91%) OXAs, 7/7 (100%) ESBL and 3/5 (60%) non-ESBL/non-CRAB (Figure 1C). Sulbactam enhanced activity by avibactam was confirmed in selected strains by gradient diffusion method (*E*-test) with commercial sulbactam strips (Liofilchem) supporting the previous observation (Figure 2).

Among OXA-producers, only one *A. baumannii* OXA-23 (M15094/AMA116) showed identical MIC values for sulbactam alone and sulbactam/avibactam (MIC 16 mg/L). Other 8 OXA-producers (7 *A. baumannii* and 1 *A.* sp. genomospecies 10) exhibited a 1 dilution decreased in sulbactam MIC by avibactam: 3 isolates already susceptible to sulbactam alone (OXA-58) and 5 resistant isolates (OXA-23).

### 2.2. Sulbactam/Relebactam Susceptibility Testing of Acinetobacter spp. Clinical Strains

With the sulbactam/relebactam combination, 73/187 (39%) *Acinetobacter* spp. isolates were inhibited by 4 mg/L (Figure 1D). The difference in activity with avibactam was due to a smaller proportion of OXA producers (52/116, 45%) inhibited with the combination of sulbactam/relebactam (Figure 1E). All 9 *Acinetobacter* isolates producing class D β-lactamases that were not inhibited by avibactam/sulbactam were also resistant to avibactam/relebactam. The MICs of sulbactam/relebactam also showed a bi-modal distribution with MIC_50_ and MIC_90_ of sulbactam/relebactam of 8 and 16 mg/L for OXA producers compared to 16 and 64 mg/L for sulbactam alone. Among NDM producers, the MIC_50_ values were 64 mg/L for sulbactam/relebactam alone compared to 256 mg/L for sulbactam.

For strains harboring MBLs, colistin and tigecycline were the only tested comparators with uniform in vitro activity (100% and 94% susceptible, respectively). Only 18% of NDM producers were susceptible to amikacin. Conversely, comparators among OXA producers were less active than the sulbactam/avibactam combination: 86% and 85% of susceptibility to colistin and tigecycline, respectively. Ceftazidime was only active against non-ESBL/non-CRAB isolates, while ceftazidime/avibactam was not active against CRAB and showed a modest 14% susceptibility against ESBL producers. Imipenem was uniformly active against non-CRAB isolates, but only 2% of CRABs (all OXA-58 producers) were susceptible to this carbapenem.

### 2.3. Sulbactam/Avibactam Susceptibility Testing of A. baumannii Knockout Strains

The potentiation of sulbactam activity by avibactam was observed in carbapenemases refractory to DBOs inhibition, as OXAs commonly found in *Acinetobacter*, suggesting a mode of action in these species unrelated to the reported inhibitory capacity. To reveal the potential sulbactam/avibactam targets, twenty-three *A. baumannii* AB5075 knockout strains were tested to identify potential sulbactam/avibactam targets. Knockout mutant in genes related with efflux pumps, two components system, cell wall synthesis genes, among others, were used. *A. baumannii* AB5075 MIC to sulbactam was 24 mg/L and showed a 16 times reduction in sulbactam MIC by addition of avibactam (Table 1).

The results observed with *A. baumannii* AB5075 mutants can be classified into three large groups.

i. Mutations affecting the activity of sulbactam alone: when comparing sulbactam MIC in the wild-type strains and isogenic mutants, a decrease in MIC values from 24 to 3 mg/L was observed in the AB5075Δ*mrcB* and AB5075Δ*mreB*, suggesting a potential role in sulbactam susceptibility (interaction). The *mrcB* and *mreB* genes, which code for a transglycosylase-transpeptidase and a cytoskeletal protein, respectively, were previously reported as genes involved in the formation of the peptidoglycan synthesis. Potentiation of sulbactam activity by avibactam is also lost in these mutant strains. Additionally, the *A. baumannii* AB5075ΔPBPG MIC of sulbactam is 1.5-fold lower than that measured for *A. baumannii* AB5075. PBPs are known to be sulbactam targets [36]. Previous reports demonstrated that the antibacterial activity of sulbactam was mediated through inhibition of PBP1 and PBP3.
ii. Mutations that result in loss of avibactam enhancement of sulbactam activity: a 1 or 0-fold decrease in the MIC values comparing sulbactam with sulbactam/avibactam MICs was observed in seven *A. baumannii* knockout strains (AB5075Δ*bfmR*, AB5075Δ*PBPG*, AB5075Δ*advA*, AB5075Δ*adeB*, AB5075Δ*mrcB*, AB5075Δ*xerD*, and AB5075Δ*mreB*). These results identified potential sulbactam and/or sulbactam/avibactam targets (Table 1).
iii. Mutations that do not affect susceptibility to sulbactam or potentiation of sulbactam by avibactam: ≤2-fold decrease in the MIC was observed in twelve *A. baumannii* AB5075 knockout strains comparing sulbactam with sulbactam/avibactam MIC. We can rule out that these mutated genes are involved in the mechanisms of action (or interaction) of sulbactam and avibactam.

## 3. Discussion

Infections with the hospital-acquired bacterium *Acinetobacter* are extremely difficult to treat. While the development of new antibiotics remains one option in the fight against strains resistant to those currently available, an attractive (and faster) alternative is the development of adjuvant therapies to restore the efficacy of existing antibiotics. A preliminary in vitro study has shown promising results with avibactam adjuvant approaches combined with sulbactam [31]. In this work we contributed new knowledge to support sulbactam/avibactam interaction.

We observed that the addition of DBOs restores sulbactam susceptibility. This interaction between DBOs and sulbactam presented the following characteristics: (i) was more potent in serine carbapenemase-producing strains, such as oxacillinases, but it was also manifested to a lesser extent in NDM-producing strains; (ii) was not related to the DBO concentration as a dose–response relationship was not observed in MICs when both DBOs were tested using twice the recommended concentration (8 mg/L) (not shown); (iv) was not related to the β-lactamase inhibitory capacity described for DBOs; and (v) avibactam was more potent in restoring sulbactam susceptibility than relebactam. The differences observed between avibactam and relebactam would support these hypotheses, the larger molecular size of the second DBO (molar mass 265.24 g/mol g·mol^−1^ vs. 348.37 g·mol^−1^, respectively) could be responsible for a reduced capability to penetrate the outer membrane and reach its targets.

Recently, ETX2514, named durlobactam, a new DBO inhibitor derived from avibactam (resulting from the addition of a double bond between C3 and C4 and a methyl group in C3 position compared to avibactam), significantly increased the susceptibility of *Acinetobacter* clinical isolates when it was combined with sulbactam [22]. Sulbactam/durlobactam has an MIC_50_/MIC_90_ of 1 and 2 mg/L against a global collection of strains. These values are comparable to those obtained in this work for the sulbactam/avibactam combination for OXA-23 producers (2 and 4 mg/L, respectively) [22]. However, the combination with durlobactam was not effective for NDM strains with MIC_50/90_ comparable to those of sulbactam alone, unlike avibactam, which showed a slight improvement in sulbactam MICs in these strains. In this context, the potent synergy observed between sulbactam and avibactam indicates that there could be different targets for each DBO.

We were unable to detect a known resistant mechanism compromising sulbactam activity. Only a small proportion of the strains presented narrow-spectrum or extended spectrum β-lactamases that affect sulbactam. No mutations in the genes coding for PBPs were observed in a previously analyzed subset of NDM producers [15]. Taken together, these results suggest that sulbactam/avibactam synergy could have an effect on other targets in MDR strains.

We explored the possible contribution in resistance to sulbactam and its interaction with avibactam of genes related with efflux pumps, two components system, cell wall synthesis genes. We observed that *mrcB* and *mreB* were two key components, in addition to the coding genes for PBPs, for phenotypic resistance to sulbactam. *mrcB*, which codes for a transglycosylase-transpeptidase, and *mreB*, which codes for cytoskeletal proteins, could play a key role in maintaining vital (or alternate) processes in wall synthesis in the presence of sulbactam, which can explain in part the observed sulbactam MIC reduction when they are inactivated. Potentiation of sulbactam activity by avibactam is also lost in these mutant strains, suggesting that conserved *mrcB* and *mreB* are a necessary (but not sufficient) prerequisite for avibactam interactions. More studies to discern the possible interplay of sulbactam and sulbactam/avibactam on *mreB* and *mrcB* are needed. We also identified key genes that seem necessary to guarantee the interaction between sulbactam and avibactam. BfmRS, a global regulatory system in *Acinetobacter*, is a key challenge for antibiotic therapy. BfmRS per se has been shown to increase the ability of *Acinetobacter* to grow in the presence of diverse antibiotics and tolerate transient, high-level antibiotic exposures [37]. As we observed, avibactam (with sulbactam) may interfere or bypass the normal protection from β-lactam toxicity mediated by BfmRS. *advA* (antibiotic susceptibility and division protein of *Acinetobacter*) is another essential gene for *A. baumannii* growth. It has been shown that reduced AdvA levels modulated antibiotic susceptibility and generate a selective pattern of hypersensitivity to fluoroquinolones and β-lactams [38]. Avibactam could be a hypothetical mediator for sulbactam hypersensitivity through *advA* interaction. Identification of other genes potentially involved in the interaction between sulbactam and avibactam, such as XerCD, tyrosine recombinases that catalyze the resolution of dimeric chromosomes [39], opens up new possibilities in target identification for designing new drug combinations.

In summary, sulbactam/DBOs, especially in combination with avibactam, demonstrated potent antibacterial activity against recent, geographically diverse clinical isolates of *Acinetobacter*, including XDR isolates such as widespread oxacillinase producers. The addition of a DBO to sulbactam appears to be a very promising strategy for managing difficult-to-treat *Acinetobacter* infections. We identified at least 6 genes that could participate in the interaction of sulbactam with avibactam. Understanding this interaction will clarify how *A. baumannii* optimizes its ability to grow under antibiotics exposition. Further extensive in vitro and in vivo studies have to be performed to confirm the potential use of these adjuvants as therapeutic alternatives.

## 4. Materials and Methods

### 4.1. Bacterial Strains

A representative panel of 187 ESBL-producers and CR *Acinetobacter* spp. clinical strains were used to test sulbactam or sulbactam in combination with avibactam or relebactam (Table S1). Among the included strains 152 were *A. baumannii*, including previously well-characterized strains such as AB5075, AB0057 and ABUH702 [40,41,42], and 35 corresponded to non-*baumannii* species. Among these isolates, 4 were non-ESBL/non-CRAB, 7 were ESBL producing, while 176 were carbapenem-resistant strains. The ATCC 17,978 *A. baumannii,* ATCC 700,903 *Klebsiella pneumoniae,* ATCC 25,922 *Escherichia coli,* and ATCC 35,218 *E. coli* strains were used as control. In addition, 23 mutant strains from Manoil Lab (Washington, DC, USA) were used to test sulbactam/avibactam activity. Among them we selected AB5075Δ*adeR*, AB5075Δ*bfmR*, AB5075Δ*bfmS*, AB5075Δ*PBPG*, AB5075Δ*advA*, AB5075Δ*adeB*, AB5075Δ*ampC*, AB5075ΔOXA-69, AB5075Δ*adeA*, AB5075Δ*mrdB*, AB5075Δ*mltB*, AB5075Δ*mrcB*, AB5075Δ*adeK*, AB5075Δ*recA*, AB5075Δ*xerD*, AB5075Δ*xerC*, AB5075Δ*lpxB*, AB5075Δ*mreB*, AB5075Δ*mreC*, AB5075Δ*mreD*, AB5075Δ*ElsL*, AB5075Δ*dnaK*, AB5075Δ*dksA*.

### 4.2. Antimicrobial Susceptibility Assay

MICs against sulbactam (range 0.12–512 mg/L), sulbactam plus 4 mg/L avibactam (range 0.12–64 mg/L) and sulbactam plus 4 mg/L relebactam (range 0.12–64 mg/L) were performed using the reference methods agar dilution according to CLSI Standards [43]. Because breakpoints are available for sulbactam alone, 4 mg/L was applied for this analysis based on the CLSI susceptible breakpoint of 8/4 mg/L for ampicillin/sulbactam for *Acinetobacter* spp. [44,45]. The in vitro activity of other comparators, such as imipenem, colistin, tigecycline, amikacin, ceftazidime (alone and in combination with 4 mg/L of avibactam) were also evaluated by dilution methods at one facility.

## Figures and Tables

**Table 1:** Sulbactam MICs against knockout *A. baumannii* strains using Mueller–Hinton agar with and without avibactam supplementation.

Strains	MH	MH + AVI 4 mg/L
AB5075	24	1.5
AB5075Δ*mrcB*	3	1.5
AB5075Δ*mreB*	3	1.5
AB5075Δ*PBPG*	8	8–12
AB5075Δ*bfmR*	24	12
AB5075Δ*xerD*	16	8
AB5075Δ*advA*	16	16
AB5075Δ*adeB*	16	16
AB5075Δ*xerC*	18	8
AB5075Δ*bfmS*	16	6
AB5075Δ*adeA*	24	8
AB5075Δ*lpxB*	24	8
AB5075Δ*mreD*	24	8
AB5075Δ*dksA*	24	8
AB5075Δ*recA*	32	12
AB5075Δ*adeK*	24	6
AB5075Δ*ampC*	24	6
AB5075ΔOXA-69	16	4
AB5075Δ*mreC*	16	4
AB5075Δ*mrdB*	16	4
AB5075Δ*mltB*	16	1.5–3
AB5075Δ*ElsL*	24	4
AB5075Δ*dnaK*	24	4
AB5075Δ*adeR*	32	3

## Data Availability

Not applicable.

## Supplementary Materials

The following are available online at https://www.mdpi.com/article/10.3390/antibiotics10050577/s1, Table S1: Features of *Acinetobacter* isolates.
